# Touchable cell biophysics property recognition platforms enable multifunctional blood smart health care

**DOI:** 10.1038/s41378-021-00329-z

**Published:** 2021-12-08

**Authors:** Longfei Chen, Yantong Liu, Hongshan Xu, Linlu Ma, Yifan Wang, Le Yu, Fang Wang, Jiaomeng Zhu, Xuejia Hu, Kezhen Yi, Yi Yang, Hui Shen, Fuling Zhou, Xiaoqi Gao, Yanxiang Cheng, Long Bai, Yongwei Duan, Fubing Wang, Yimin Zhu

**Affiliations:** 1 Affiliations School of Physics & Technology, Key Laboratory of Artificial Micro/Nano Structure of Ministry of Education, Wuhan University, Wuhan, 430072 China; 2 Shenzhen Research Institute, Wuhan University, Shenzhen, 518000 China; 3 Department of Hematology, Zhongnan Hospital, Wuhan University, Wuhan, 430071 China; 4 Department of Laboratory Medicine, Zhongnan Hospital, Wuhan University, Wuhan, 430071 China; 5 Remin Hospital of Wuhan University, Wuhan University, Wuhan, 430060 China; 6 School of Medicine, Zhejiang University, Hangzhou, Zhejiang, 310002 China

**Keywords:** Micro-optics, Engineering, Optical sensors

## Abstract

As a crucial biophysical property, red blood cell (RBC) deformability is pathologically altered in numerous disease states, and biochemical and structural changes occur over time in stored samples of otherwise normal RBCs. However, there is still a gap in applying it further to point-of-care blood devices due to the large external equipment (high-resolution microscope and microfluidic pump), associated operational difficulties, and professional analysis. Herein, we revolutionarily propose a smart optofluidic system to provide a differential diagnosis for blood testing via precise cell biophysics property recognition both mechanically and morphologically. Deformation of the RBC population is caused by pressing the hydrogel via an integrated mechanical transfer device. The biophysical properties of the cell population are obtained by the designed smartphone algorithm. Artificial intelligence-based modeling of cell biophysics properties related to blood diseases and quality was developed for online testing. We currently achieve 100% diagnostic accuracy for five typical clinical blood diseases (90 megaloblastic anemia, 78 myelofibrosis, 84 iron deficiency anemia, 48 thrombotic thrombocytopenic purpura, and 48 thalassemias) via real-world prospective implementation; furthermore, personalized blood quality (for transfusion in cardiac surgery) monitoring is achieved with an accuracy of 96.9%. This work suggests a potential basis for next-generation blood smart health care devices.

## Introduction

Label-free disease diagnosis and quality monitoring by natural biophysics properties of cells is expected to be significant for the future of smart health care and point-of-care (POC) applications^1–5^. For example, as a typical and practical diagnostic indicator, blood cell morphology is widely used for blood testing^6,7^. However, complex equipment, well-trained operation, and dyes are often required to achieve high diagnostic accuracy for cell morphology-assisted blood diagnosis^8–10^ because of the inhomogeneity and inconsistency of cell morphological transition dynamics^11–13^. Cell deformability serves as an important indicator of the real-time response to cell status. However, monitoring cell deformability has not been applied to the field of POC blood smart health care due to complex equipment requirements and associated operational difficulties^14–22^.

Optofluidics^23,24^ are characterized by high integration^20,25–28^, precise optical manipulation^29–31^, and portability^32,33^, and they provide a good platform for studying cell biophysics properties. Currently, a series of good works have been reported for cell deformability monitoring, which implies a potential clinical application for POC blood cell analysis^34,35^. Here, we introduce a smart optofluidic engineering platform for multifunctional blood testing via precise cell biophysics property recognition both in mechanics and morphology with high accuracy, low cost, and ease of operation. Based on the elastic strain and stress coupling of the hydrogel, blood cell deformation is achieved by manipulating a hydrogel actuator using a mechanical transfer device. A tunable imaging platform (depth of field: 10 µm) was designed for capturing blood cell images at different focusing surfaces. Images, morphologic (diameter, circularity, axis ratio, and corresponding distribution width), and mechanical (deformability and distribution width) parameters of blood cells were obtained via a developed image algorithm employed on a smartphone^36–39^. The integrated data were then used as input for cloud computing, and they were then transformed into vector tables and loaded into image vectors to perform pathological diagnosis and quality monitoring based on a trained neural network^40,41^. The strong system performance was validated with clinical samples, which provides a new strategy for future POC blood smart health care.

## Results

### Cell deformation via photocurable hydrogel actuator manipulation

**Fig. 1:**
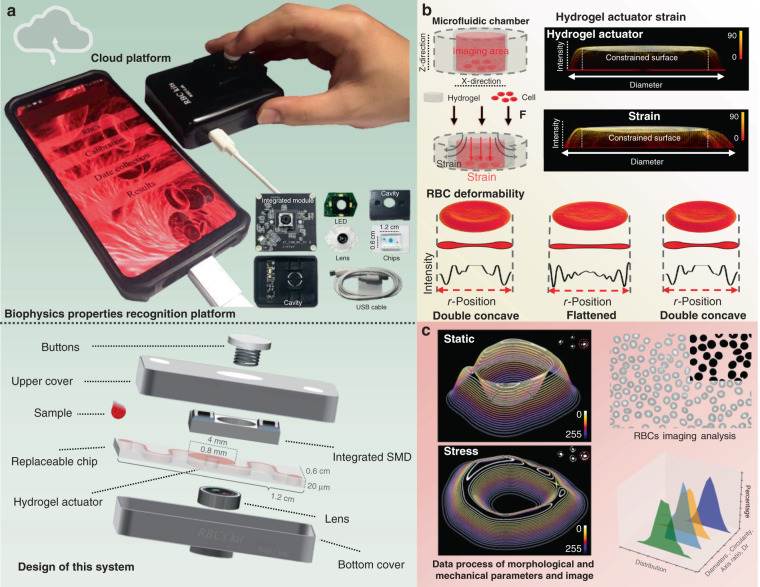
Process flow for biophysics properties collected and analyzed of RBCs based on this device. **a** Device design. **b** Elastic deformation of photocurable hydrogel actuator, and the cell strain and recovery process. **c** Date collection and analysis performed on the smartphone

**Fig. 2:**
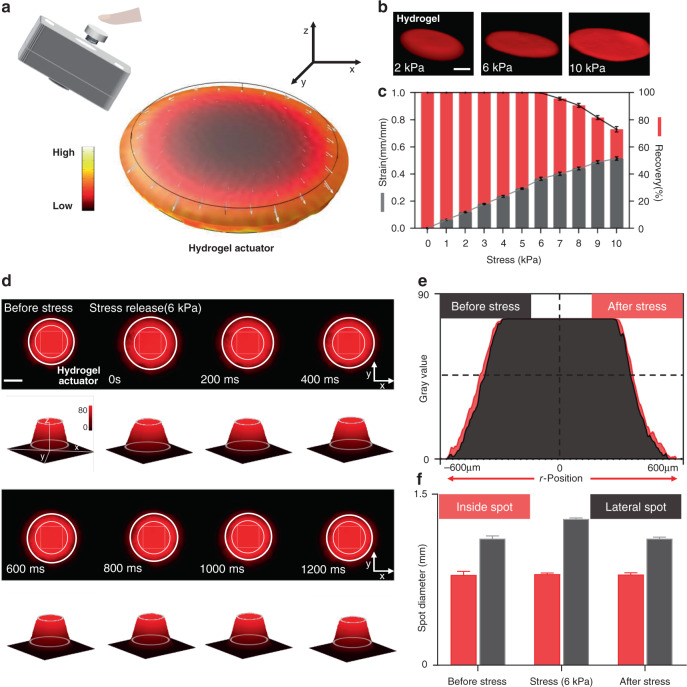
Photocurable hydrogel actuator manipulation. **a** The developed model of the stress and the strain for the photocurable hydrogel actuator. **b** Three-dimensional confocal micrographs of photocurable hydrogel under different stresses, scale bar is 300 μm. **c** The stress–strain and stress-recovery histograms of the photocurable hydrogel actuator. **d** The deformation–relaxation fluorescence micrographs of the photocurable hydrogel actuator and the three-dimensional light intensity distribution of the light spot, scale bar is 500 μm. **e** The light intensity distribution map of the photocurable hydrogel actuator before and after stress. **f** The inside and lateral spot analysis histograms of the photocurable hydrogel actuator under multiple compression measurements

**Fig. 3:**
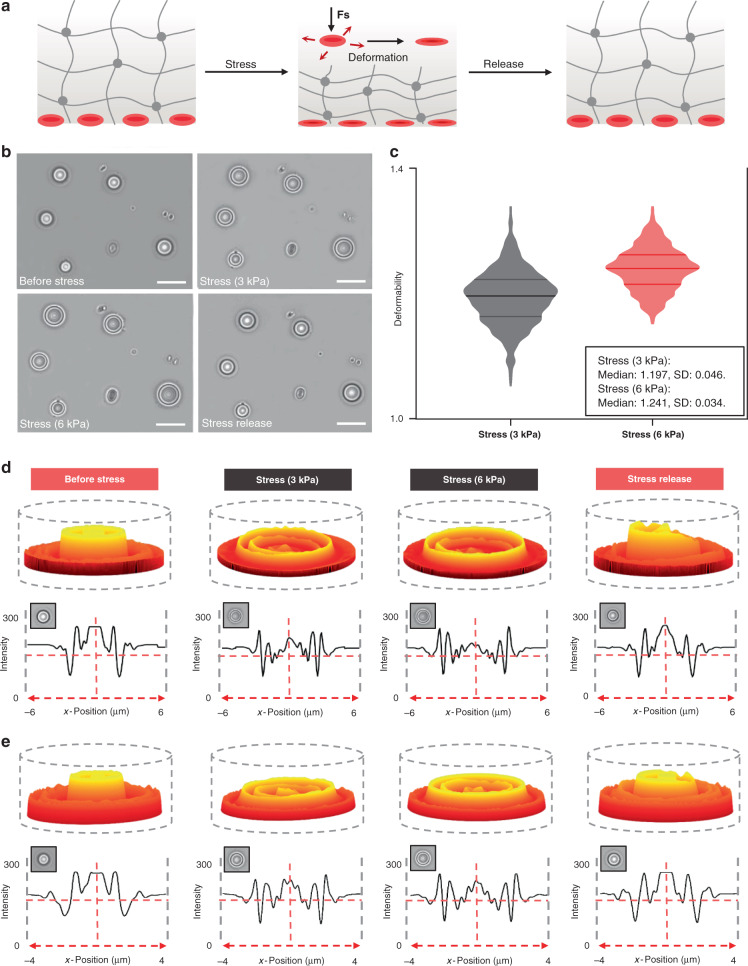
RBCs deformation via hydrogel actuator manipulation. **a** Schematic of RBCs deformation caused by longitudinal shrinkage of the hydrogel mesh. **b** Micrographs of RBCs under different stresses and recovery, scale bar is 10 μm. **c** Scatter dot plot of RBCs deformability under different stress (median with SD). **d**, **e** Three-dimensional light intensity distribution diagram and light intensity distribution diagram of the two-dimensional section (*y* = 0) for a single RBC

The developed optofluidic chip integrated with a flexible photocurable hydrogel actuator is shown in Fig. 1. Finger pressure was coupled to the elastic hydrogel through a mechanical transfer device, which deformed the hydrogel, and it enabled precise control of the pressure applied to the hydrogel actuator by continuous pressure for stable deformation image acquisition. Through model development, the strain distribution was obtained by coupling the pressure to the top part of the hydrogel actuator. The results indicated that the hydrogel compressed vertically and expanded horizontally under the pressure coupling action. The hydrogel actuator compressed in the vertical direction, and an insignificant expansion of the constrained surfaces (top and bottom surfaces) was observed in the lateral direction (Fig. 2a). Elastic and plastic deformations (reversible and irreversible deformations) of the hydrogel occurred under different pressures. The irreversible hydrogel deformation leads to irregular spatial grid shrinkage and expansion, which interferes with cell deformation measurement. We measured the hydrogel stress–strain curve in a microfluidic chamber. The hydrogel precursor (10 w/v% Gelma solution, see Methods) was labeled with rhodamine 6G (Sigma), and a red fluorescently labeled hydrogel actuator was photocured and cross-linked in the microfluidic cavity with blue light irradiation (405 nm, 10 s) under a Filin photomask. Different pressures (1–10 kPa) were applied to the hydrogel actuator. With an increase in pressure, the hydrogel changes from regular to irregular deformation (Fig. 2b). Figure 2c shows a histogram of the stress–strain and the stress recovery of the hydrogel. The results indicate that hydrogel elastic deformation occurs when the stress is in the range of 1–6 kPa. When the stress is greater than 6 kPa, it causes hydrogel elastic deformation. The hydrogel actuator (*d* = 800 µm, *h* = 20 µm) deformation–relaxation test was conducted using a confocal microscope (Nikon, A1R). The deformation–relaxation fluorescence distribution maps of the hydrogel are recorded and analyzed in Fig. 2d. The internal spot refers to the constrained surface of the hydrogel (the interface between the hydrogel and the substrate, the imaging region), while the lateral spot refers to the lateral deformation due to the hydrogel stress. As shown in the three-dimensional light intensity distribution plot of Fig. 2d (the upper white circle is the inner light spot, and the lower white circle is the outer light spot), the cylindrical shape of the three-dimensional light intensity distribution is hydrogel residue on the surface based on the flashlight photocuring process, which does not interfere with the overall hydrogel actuator deformation. The results show that the inner spot remains stable (spot size: 800 μm, gray value: 76.9) in the hydrogel elastic strain. The outer optical spot undergoes expansion and then reverts upon withdrawal of the stress. This is a direct indication that the constrained surface of the hydrogel actuator maintains excellent stability during strain. The intensity distribution map (Fig. 2e) further verified that the hydrogel can be recovered after elastic deformation. Next, multiple compression measurements (Fig. 2f) were conducted, and the internal speckle size remained stable during the compression process (mean = 807, *σ* = 4.345 SDs, and *n* = 60). These results indicate a basis for cell imaging and deformation in flexible photocurable hydrogel actuators. The longitudinal hydrogel (phosphate-buffered solution (PBS)-filled, blood sample: hydrogel precursor = 1: 100) grid was compressed, forcing the red blood cells (RBCs) in the grid to deform regularly with stress (Fig. 3a). Then, the stress deformability of RBCs was measured, and stresses of 3 and 6 kPa were applied to the hydrogel actuator. Figure 3b indicates that RBCs deformed regularly with a hydrogel strain, and the uniform RBC orientation (double concave side in *Z*-direction) was based on the settling process. A total of 2362 RBCs were randomly selected to measure the deformation rates of 3 and 6 kPa. Figure 3c shows that the average RBC deformation rates between 3 and 6 kPa were 1.194 (*σ* = 0.045) and 1.241 (*σ* = 0.033), respectively, which indicates that when the RBCs accept higher stress, the deformability is larger and the range of variation becomes narrower. RBCs undergo internal osmotic pressure and the cell membrane morphology changes during the deformation process. To study the morphological changes in the cell membrane in the RBC process, the light intensity distribution was analyzed before and after RBC deformation. Furthermore, as shown in Fig. 3d, e, the RBCs changed from a biconcave shape to spherical, and the middle concave structure tended to flatten as the strain gradually increased and the cell membrane expanded in the XY plane. When the stress was released, the RBCs returned to the double concave structure. The light intensity distribution diagram of the two-dimensional section (*y* = 0) directly illustrates the RBC deformation and recovery process in this system.

### Cell biophysics properties collected and analyzed in the smartphone

**Fig. 4:**
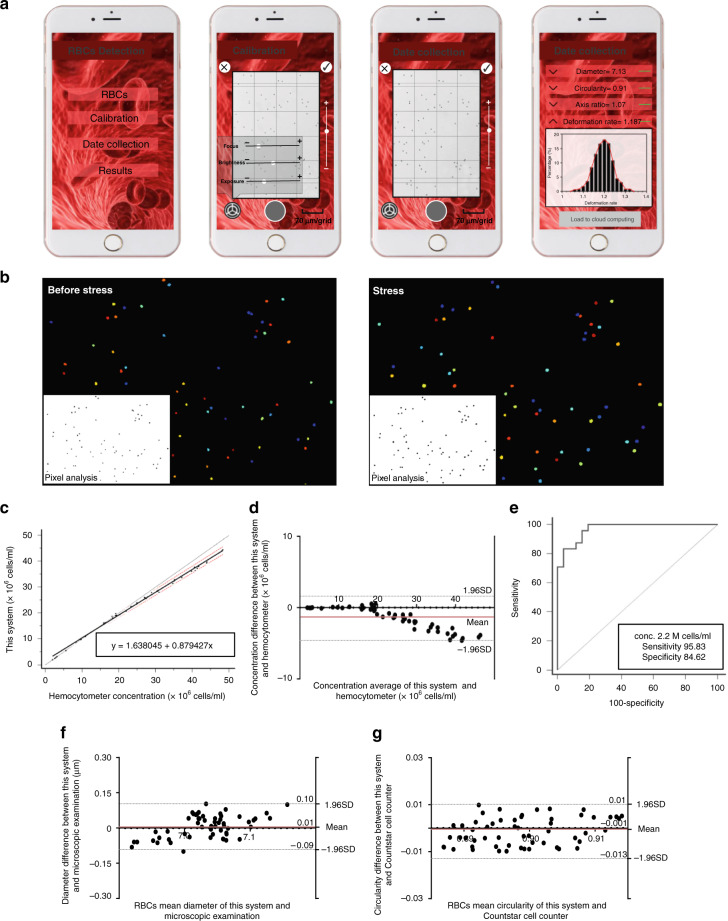
Data collected and analyzed in the smartphone and the performance of this system. **a** The interface of the smartphone for data collection and analysis. **b** Program algorithm for the statistical analysis of cell number, area, and perimeter before and after stress. **c** Direct comparison of RBCs concentrations calculated by the smartphone-based device and standard hemocytometer. The solid black line represents the regression line, the solid gray line represents the identity line, and two dashed red lines represent the confidence band (*n* = 50). **d** Bland–Altman analysis to compare the results obtained by the smartphone-based device and standard hemocytometer. The red dashed line is the mean difference of the methods, and the gray dashed lines represent the 95% LOAs. **e** ROC curves. **f**, **g** Bland–Altman analysis to compare the mean diameter, circularity obtained by the smartphone-based device and microscopic examination, and Countstar cell counter (*n* = 56)

Figure 4a shows the operating interface of the smartphone data acquisition and analysis process and the cell imaging before and after deformation realized by a miniaturized tunable optical imaging system (field of view (*H* × *V*, mm): 1.81 × 1.02, resolution: 1 µm)^39^. The image processing steps are illustrated in Fig. 4b, and the developed image algorithm was used to identify primary cells and remove stacked cells. Furthermore, the morphologic (diameter, circularity, axis ratio, and corresponding distribution width) and mechanical (deformability and distribution width) blood cell parameters were obtained through imaging analysis (see “Methods”). We used a double-blind approach wherein 50 healthy RBC samples were measured based on this system, and a parallel count was performed by a technician using a standard hemocytometer (Nexcelom cellometer, America) to evaluate the system performance in terms of counting samples based on changes in the concentrations in the lower range. Figure 4c indicates that the linearity of the two methods has no obvious deviation (*P* = 0.07). Based on the Passing–Bablok regression analysis (*n* = 50), the A intercept value was 1.6380 with a confidence interval (CI) of 1.0959–2.1988, and the B slope value was 0.8794 with a CI of 0.8587–0.8977. Bland–Altman analysis (*n* = 50) was used to compare this intelligent system, and the manual RBC count showed a mean bias of −1.492 million RBCs/mL with a standard deviation (SD) of 1.57 × 10^6^ RBCs/mL (Fig. 4d). The limits of agreement (LOAs) ranged between −4.564 and 1.581 million RBCs/mL. These statistical analyses indicated that the difference between the hemocytometer and the proposed system increased as the RBC concentration increased in the samples. Receiver operating characteristic (ROC) analysis was performed to establish a threshold for optimized sensitivity and specificity (as shown in Fig. 4e), and the ROC analysis results revealed that the RBC concentration threshold of 2.2 × 10^6^ RBCs/mL yields a sensitivity of 95.83% (CI: 78.9–99.9) and specificity of 84.62% (CI: 65.1–95.6) for this system to count RBC samples; furthermore, the area under the curve (AUC) was 0.970 with CI ranging between 0.877 and 0.988. Bland–Altman analysis (*n* = 56) was performed to evaluate the imaging/analysis system performance in the diameter, axis ratio, and circularity analyses of healthy RBC samples from the hospital compared to the expert microscopic examination and Countstar cell counter. As shown in Fig. 4f, the mean bias was −0.6664% with an SD of 4.84% for the mean diameter (LOA: −0.088 to 0.102), and the mean bias was −0.0769% with an SD of 1.25% of the axis ratio (Fig. S6, LOA: −0.025 to 0.024). The results showed an insignificant difference between the microscopic examination and smartphone-based device for measuring the diameter and axis ratio. Furthermore, we performed Bland–Altman analysis of RBC samples with mean circularity (i.e., SD, Fig. 4g). The mean bias was −0.14% with an SD of 0.59% of the distribution width (LOA: −0.013 to 0.010). Collectively, the Bland–Altman analyses showed an insignificant difference between the Countstar cell counter and the smartphone-based device in measuring circularity.

**Fig. 5:**
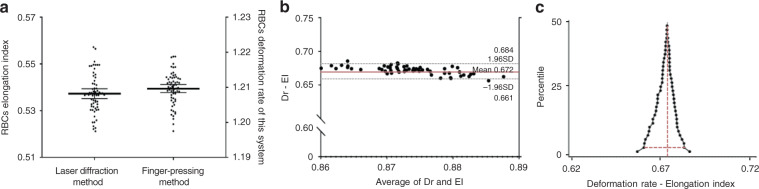
Performance of this device for RBCs deformability monitoring. **a** Scatter dot plot of RBCs deformability based on this system and the laser diffraction method (mean with SD). **b** Bland–Altman analysis to compare the results obtained by the finger-pressing method and the laser diffraction method. The red dashed line is the mean difference of the methods, and the gray dashed lines represent the 95% LOAs. **c** The mountain plot analysis computed a percentile for each ranked difference between the laser diffraction method and the finger-pressing method for cell deformability, the vertical red dashed line represents the center of the mountain, and the horizontal red dotted line represents the 5th to 95th percentile

Figure 5a shows a scatter plot of the RBC elongation index value (EI, mean = 0.5373, *σ* = 0.0082) based on the results of a laser optical rotational red cell analyzer (Lorrca, shear stress = 6 Pa) and RBC deformability value (Dr, mean = 1.210, *σ* = 0.0046)-based finger-pressing method obtained via a mechanical transfer device (Stress = 3 kPa) for 63 healthy RBC samples. We conducted Bland–Altman analysis and mountain plot analysis to analyze the performance and accuracy of the finger-pressing method for cell deformability. The Bland–Altman analysis (*n* = 63) results comparing the finger-pressing method and laser optical rotational red cell analyzer indicated a mean bias of 0.6723 with an SD of 0.0059 (Fig. 5b). The LOAs ranged from 0.6607 to 0.6840. The mountain plot analysis computed a percentile for each ranked difference between the human-capillary-like microchannel method and the finger-pressing method for cell deformity. To obtain a folded plot, the following transformation was performed for all percentiles above 50: percentile = 100 − percentile. These percentiles are then plotted against differences between the two methods. As shown in Fig. 5c, the mountain is centered at 0.6731 with 0.6607 (5th percentile) to 0.6818 (95th percentile). These statistical analyses showed that the finger-pressing method corresponded well with a laser optical rotational red cell analyzer for cell deformity monitoring.

### Artificial intelligence-based modeling of cell biophysics properties related to blood diseases

**Fig. 6:**
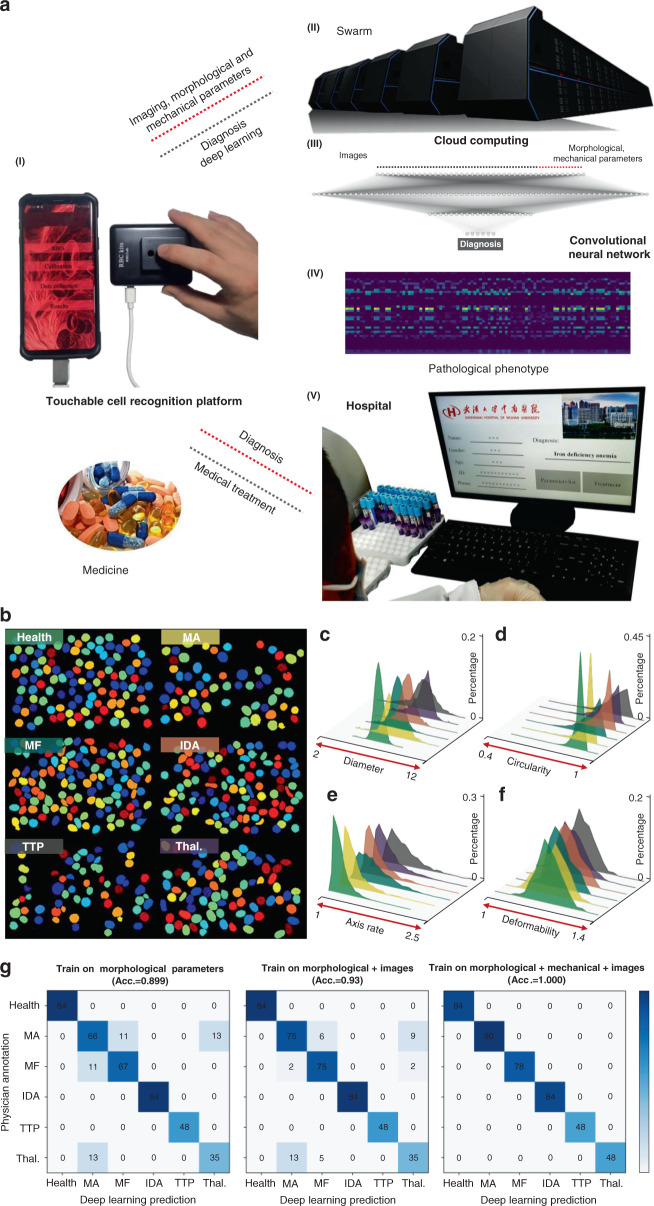
Artificial intelligence-based modeling of cell biophysics properties related blood diseases. **a** I The finger-press type microfluidic/smartphone imaging system collects the integrated images, morphological, and mechanical parameters loaded into cloud computing. II The pathological diagnosis is performed via cloud computing based on deep learning. III The three fully connected layers contain 40, 64, and 20 vectors, respectively, of which the first fully connected layer contained 32 image vectors and 8 morphological and mechanical parameter vectors. IV Feature extraction of the multiparameter training models in 432 clinical samples. V Diagnostic reports are transmitted to the physician via the cloud sharing platform. **b** Identification of the primary objective for health, MA, MF, IDA, TTP, Thai. blood samples. **c**–**f** Distributions of diameter, circularity, axis ratio, and deformability of the samples. **g** Confusion matrices comparing the performance on three training pathological diagnosis models between expert diagnosis and deep learning

Figure 6a shows the interactive design of this optofluidic POC (OPOC) system. The smartphone (Fig. 6a I) collected the images and variables (morphological: diameter, circularity, axis ratio, and corresponding distribution width; mechanical: deformability and distribution width) integrated into the cloud (Fig. 6a II) based on a trained neural network to perform the diagnosis. The deep learning system has two main parts: a variable number of deep convolutional neural network modules for processing a flexible number of input images and a module for processing multiple variables. The trained neural network contained six convolutions and three fully connected layers. As shown in Fig. 6a III, the 3 fully connected layers contained 40, 64, and 20 vectors, of which the first fully connected layer contained 32 image vectors and 8 variable vectors (Fig. 6a IV, feature extraction of participants). The diagnostic data are then returned to the smartphone; the patient can share the diagnosis with the detailed parameters with the doctor via cloud sharing to allow the doctor to provide the next steps with regard to the medication or further hospital treatment (Fig. 6a V).

We performed neural network training for five typical clinical blood diseases, including megaloblastic anemia (MA), myelofibrosis (MF), iron deficiency anemia (IDA), thrombotic thrombocytopenic purpura (TTP), and thalassemia (Thal.). Through cell imaging and analysis (Fig. 6b)^36^, the morphological and mechanical parameters are shown in Fig. 6c–f. Toward that end, we collected a total of 432 samples (Fig. S7), including 84 healthy, 90 MA, 78 MF, 84 IDA, 48 TTP, and 48 Thal. from participants. Then, diagnostic models based on deep learning were conducted. To develop and validate the system, we used a random segmentation approach for blood disease cases: 90% of the cases were used for development and 10% for validation. Multiple training and validation cycles were executed, wherein the development and training sets were randomly disrupted in each cycle. The reference standard for each case was determined by the aggregated opinion of the hematologist who independently tested the cases (see Methods). Figure 6g shows the performance of the three trained neural networks. The diagnostic model that combined the image, morphological, and mechanical parameters achieved 100% diagnostic accuracy for the 6 classifications (*n* = 432); furthermore, the diagnostic accuracy was consistent with the examination conducted by professional physicians, thereby indicating a potential method via cell multirecognition to further improve diagnostic accuracy.

### Personalized blood-quality monitoring

**Fig. 7:**
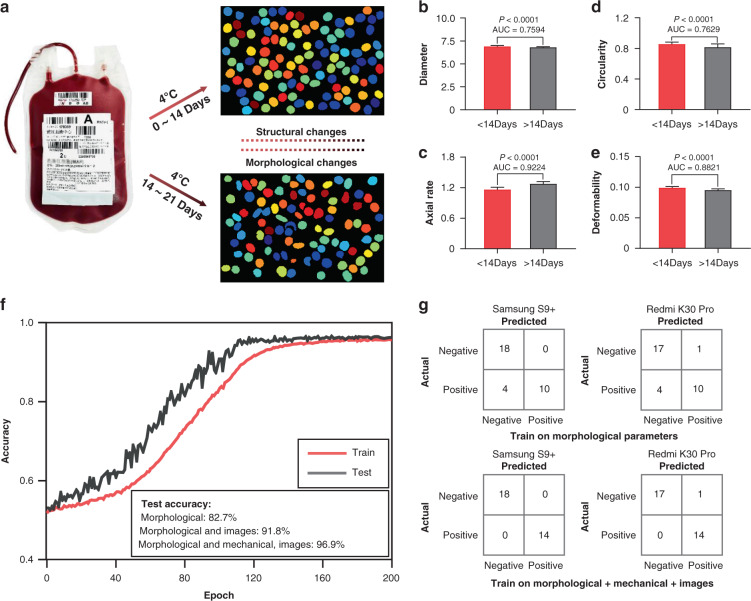
Artificial intelligence-based modeling of cell biophysics properties related storage lesion. **a** The diagram of RBCs morphology and structural changes during storage. **b**–**e** The statistical histogram of RBCs diameter, circularity, axis ratio, deformability between 0–14 days’ storage and 14–21 days (*n* = 98). **f** Diagnostic accuracy of three training models. **g** Confusion matrices comparing performance on different smartphones (actual was defined that tested by point-of-care device for Community Health Service Centers) between morphological and multiparameters training model

RBCs undergo progressive structural and functional changes during storage (Fig. 7a), and the development of personalized blood transfusion quality monitoring for complex blood transfusion needs will help improve the blood utilization rate. Previous studies proposed a hypothesis that serious complications and mortality after cardiac surgery increased when transfused RBCs were stored for more than 2 weeks^42^. Based on this system, we performed blood-quality monitoring of 98 cryopreserved RBC (4 °C) samples during storage. Among them, 48 RBC samples were cryopreserved within 14 days, and 50 were cryopreserved for more than 14 days (14–21 days). The results showed that there was no significant statistical correlation between RBC counts in the two intervals, and the *P* value was 0.1105, whereas a significant statistical correlation was observed in the diameter, circularity, axis ratio, and deformability. The corresponding AUCs were 0.7594 (CL: 0.6651–0.8537), 0.7629 (CL: 0.6703–0.8556), 0.9224 (CL 0.8819–0.9769), and 0.8821 (CL: 0.8194–0.9448) (Fig. 7b–e). Then, neural network training and testing of the blood-quality monitoring model were conducted, and the results showed that the proportion of misdiagnosed samples was as high as 17.3% when the neural network was trained with morphological parameters. When the diagnostic model combined the morphology parameters and the image, the proportion of misdiagnosed samples decreased to 8.2%. After adding mechanical parameters along with training, the diagnostic accuracy of the deep learning system reached 96.9% (Fig. 7f). Misdiagnosis samples were concentrated near the separation points, and there were no significant differences in the morphological and mechanical properties (Table S3). Figure 7g shows the system performance based on various smartphone-based quality monitoring of 32 cryopreserved RBC (4 °C) samples during storage. Among them, 18 RBC samples were cryopreserved within 14 days, and 14 RBC samples were cryopreserved for more than 14 days (14–21 days). All samples were tested in parallel with both Samsung S9+ and Redmi K30 Pro, and the results show that the system correctly classified 100% (0 of 32) of the Samsung S9+ data and 96.87% (1 of 32) of Redmi K30 Pro data.

## Discussion

This paper introduces an innovative biophysics property recognition platform that enables multifunctional POC blood smart testing. The comparison of this system diagnostic performance among experts in five clinical blood diseases via real-world prospective implementation (*n* = 432, 100% accuracy) and the personalized assessment of the stored blood quality (*n* = 98, 96.9% accuracy) demonstrated the feasibility of all key functional aspects as well as the practical utility of this system in the smart health care field. Furthermore, this smart optofluidic system can be extended to disease diagnosis accompanied by abnormal morphology and mechanics of blood cells, etc.; it can also be used to evaluate the recovery of patients with blood diseases after treatment. Moreover, this system can be expanded into in vitro cultured cell quality monitoring of sperm, oocytes, follicles, etc., and provides a potential platform for further drug and target screening.

For the precise recognition of cellular morphology and mechanics, a steady fluid-assisted imaging platform (three-dimensional hydraulic focusing) and precise mechanical manipulation (shear force, laser diffraction, etc.) are required for the measurement; however, they have portability limitations and associated operational difficulties for untrained users. In this study, we propose a revolutionary OPOC platform that uses hydrogel actuator-assisted compression imaging to achieve precise cell recognition both in morphology and mechanics with high accuracy, low cost, and ease of operation in a miniaturized integrated system. Furthermore, this can be combined with microneedles to achieve continuous blood extraction and analyses in a chip. The developed deep learning algorithms and cloud computing enable label-free and automatic blood disease-assisted diagnosis/evaluation of blood quality. Furthermore, the construction of an ecological POC engineering system for blood (image, morphology, mechanics, and small molecules), which can expand the number of human participants and increase the range of types of typical blood diseases, deep learning-based mixed-blood disease model development will be a powerful tool in promising smart health care.

## Materials and methods

### Fabrication of the replaceable microfluidic chip

The replaceable microfluidic chip (1.2 cm × 0.6 cm × 20 µm) was fabricated using the standard soft lithography process. First, the SU8 photoresist (MicroChem, SU8-50) was dropped on the silicon wafer and rotated by a homogenizer (KW-4A) to obtain a 20 μm photoresist coating layer. After prebaking, the master was exposed to UV light under a glass/chromium mask using a mask aligner (OAI, 506). Then, a positive relief of the photoresist on the surface was generated, which was used as the mold for future replications. The 20 g polydimethylsiloxane (PDMS) prepolymer (Dow Corning, Sylgard 184) prepolymer was then poured over the master (5 in.) and stored in an oven for 1 h at 75 °C. The PDMS replica was then peeled off and sealed against a flat PDMS slab to form the microchannel after oxygen plasma bonding.

### Formation of photocurable hydrogel actuators in microfluidic chips

Ten milliliters of PBS was added to a brown bottle containing lithium phenyl(2,4,6-trimethylbenzoyl) phosphinate (LAP, EFL) and heated at 40–50 °C in the dark for 30 min, and it was shaken several times (>5 times) during this period to obtain an initiator solution. Then, 10% mass Gelma material (EFL-GM-60, Suzhou, China) was added to the initiator solution (Gelma: Initiator solution = 1:10), heated at 40–50 °C in the dark for 30 min, and shaken several times (>5 times) during the period. A 0.22-micron sterile syringe filter was used to filter and sterilize the hydrogel precursor solution immediately, storing at 4 °C afterward (storage period > 2 weeks).

For each test (Fig. S3), the blood was diluted with a hydrogel precursor (blood sample:hydrogel precursor = 1:100), and the mixed solution was injected through the chip inlet, which fills the microfluidic chamber. After standing for 2 min (Fig. S4), the RBCs precipitate to the bottom of the chip and form a hydrogel actuator (*D* = 800 µm, *H* = 20 µm) in the microfluidic chamber through blue light (Flashlight, FENIX TK25RB) exposure under a photomask (Filin film, Jixianguangdian). The data measurements need to be completed within 10 min because of the natural changes in blood cells in vitro.

### Mechanical transfer device tested by untrained users

As shown in Fig. S5, to evaluate the mechanical transfer device performance on untrained users, we randomly selected two untrained patients to perform compression on the mechanical transfer device. Each experiment was performed with three consecutive compressions for 3 s of constant force application and real-time stress monitoring with a planar pressure tester (DS2-XD, Zhiqu). The results indicated that the mechanical transfer device met the ease of operation and high stability requirements for untrained users.

### Imaging analysis

Through the tunable optical imaging system, the smartphone collected the field of view of the RBCs through autofocus. The RBC images were then converted into a grayscale image (8-bit). After the light intensity and contrast were automatically adjusted by the software, the RBC images were converted into binary images using the threshold operation. The threshold setting was used to remove the stacked cells. Then, a hole-filling operation was performed to fill the contour and improve the calculation accuracy. Finally, the morphologic and mechanical parameters were calculated via software pixel analysis. The deformation index (Dr) is defined as ${\rm{Dr}} = \sqrt {S_{\rm{d}}/S_{\rm{ud}}}$, where *S*_d_ denotes the pixel area after deformation and *S*_ud_ denotes the pixel area before deformation. Mean diameter is defined as diameter = $\sqrt {\rm{area}/\pi }$; axis ratio is defined as axis ratio = MaxFeret/MinFeret (Feret Diameter); circularity is defined as circularity = 4*π* × area/perimeter^2^; circularity = 1 indicates a perfect circle.

### Developed deep learning system

Input images were obtained after data augmentation was applied to the raw images by randomly rotating, cutting, and flipping. Then, we used AlexNet with pretrained weight coefficients on the ImageNet dataset for training. The layer before the last layer was set to have 32 neurons. After training, these vectors were extracted as feature vectors in the embedded space where the neural network learns. All inputs were resized to 224 × 224 pixels for images with a scale of 0.1 mm × 0.1 mm for cell imaging in the hydrogel actuator, and the images were cropped to the same scale—0.1 mm × 0.1 mm—before being used as an input. The 32-dimensional feature vectors extracted in images were combined with the 8-dimensional mechanical and morphological data obtained from imaging analysis and the combined feature vectors to train a neural network with two hidden fully connected layers. The dropout method was used in the training process to avoid overfitting and increase generalization performance.

### Ethics statement

All experiments were conducted in accordance with the Declaration of Helsinki and the International Ethical Guidelines for Biomedical Research Involving Human Subjects. Anonymized tissue samples were retrieved from the Hematology Laboratory patient database after obtaining approval from the Ethics Committee of Zhongnan Hospital of Wuhan University (No. 2020153).

### Hospital pathology testing after an intelligent diagnosis via real-world prospective implementation

The hospital pathological diagnosis of the patients (432 participants with this device) after an intelligent diagnosis was performed. Peripheral blood specimen smears, typical bone marrow smears, and bone marrow biopsy smears are shown in Fig. S8 for six typical cases, and Fig. S9 shows the radar map (Table S1) with morphologic and mechanical parameter distributions. The intelligent diagnostic data of 30 typical participants in 432 participants are listed in Supplementary Table S2.

### Complete blood cell test

Venous blood (2–3 mL) was placed in ethylenediaminetetraacetic acid (EDTA) anticoagulation tube, and the complete blood count test was performed on a Beckman Coulter LH750 (Beckman Coulter, Miami, Florida).

### Blood smear

Each patient took one drop of fingertip blood and quickly pushed it into a peripheral blood smear. All slides were subsequently manually stained with May-Gruenwald-Giemsa staining by the same technician, and they were visually examined by two independent experienced clinical technicians using light microscopy.

### Bone marrow smears and bone marrow biopsy

Bone marrow puncture was performed at the posterior superior iliac spine. Bone marrow fluid (0.1–0.2 mL) was slowly aspirated, and the bone marrow fluid was pushed onto the slide to quickly generate eight smears. Bone marrow smears were stained with Wright Giemsa, and immunohistochemical detection was carried out based on specific conditions. The cytoplasm and quantity of the bone marrow were observed under a microscope. The morphological analysis was performed jointly using two senior laboratory physicians.

## Supplementary information

Supplementary_Materials

Ethical approve

## Data Availability

All data needed to evaluate the conclusions in the paper are present in the paper and/or the Supplementary Materials. Additional data related to this paper may be requested from the authors.
