# Supplementary material for: Touchable cell biophysics property recognition platforms enable multifunctional blood smart health care
Source: Microsyst Nanoeng. 2021 Dec 8;7:103. doi: 10.1038/s41378-021-00329-z (PMC8651774; doi:10.1038/s41378-021-00329-z)
Supplement: Supplementary file 1 — Supplementary_Materials [file 41378_2021_329_MOESM1_ESM.docx]

Supplementary Materials for

Touchable cell biophysics properties recognition platform enables multi-functional blood smart healthcare

Longfei Chen,^1,2†^ Yantong Liu,^1,2†^ Hongshan Xu,^1^ Linlu Ma,^3^ Yifan Wang,^1^ Le Yu,^1^ Fang Wang,^1^ Jiaomeng Zhu,^1^ Xuejia Hu,^1^ Kezhen Yi,^5^ Yi Yang,^1,2*^ Hui Shen,^3^ Fuling Zhou,^3*^ Xiaoqi Gao,^1^ Yanxiang Cheng,^4*^ Long Bai,^6^ Yongwei Duan,^5^ Fubing Wang,^5^ Yimin Zhu,^6^

*Corresponding author. Email: [yangyiys@whu.edu.cn](mailto:yangyiys@whu.edu.cn)

[zhoufuling@whu.edu.cn](mailto:zhoufuling@whu.edu.cn)

yanxiangcheng@whu.edu.cn

**This PDF file includes:**

Fig. S1. Components of OPOC system and each test cost.

Fig. S2. Micrograph of the micro-particles (3 μm) based on this system.

Fig. S3. Sample extraction and photo-curable hydrogel actuators.

Fig. S4. Cell imaging plane based hydrogel; X-Z direction of the hydrogel; and real-time monitoring of settling process.

Fig. S5. Various mechanical transfer device via finger press, and mechanical transfer device tested by untrained users.

Fig. S6. Bland–Altman analysis to compare the mean axis ratio obtained by this system and microscopic examination.

Fig. S7. Distributions of diameter, circularity, axis ratio, and deformability of 84 Health, 90 MA, 78 MF, 84 IDA, 48 TTP, and 48 Thal. participants from different patient donors.

Fig. S8. Hospital pathology of six typical participants (a-f).

Fig. S9. Radar map of six typical participants with morphologic and mechanic parameters distribution.

Table S1. Patient admission pathology report of six typical participants after intelligent diagnosis.

Table S2. Mean diameter, circularity, axis ratio, and deformability of the 30 typical participants with this device.

Table S3. Mean diameter, circularity, axis ratio, and deformability of the misdiagnosis samples for blood quality.

**Supplementary Test**

**Design of the devices**

The device (Fig. S1, right) is divided into five modules including the packaging shell, optical lens assembly, microfluidic chip, built-in light source, and mechanical transfer device. The packaging shell was fabricated by 3D photo-curable (Freeformer 300-3X) based on acrylonitrile butadiene styrene (ABS), and it is divided into two parts: the bottom part provides the optical lens assembly (groove structure) and the microfluidic chip module (Clamp structure, 3.4 cm × 2.4 cm), and the top part contains a finger-pressure mechanical transfer device (hole structure, R = 0.6 cm) and a patch power supply (Angjie polymer lithium battery, 12 mm × 10 mm × 3 mm, 40 mAh). The integrated optical lens assembly was a microscope (resolution: 1 µm, H × V: 1.81 mm × 1.02 mm, working distance: 0.75 mm, KENWEIJIESI). The built-in light source contains two surface-mounted device (SMD) LEDs (white 460 nm), which provide sufficient light intensity at high magnification. The micrograph of the micro-particles (3 µm) based on this system is shown in Fig. S2. The mechanical transfer device consists of round buttons (R = 0.65 cm, Freeformer 300-3X), springs (K = 5 N/cm, Lee Spring), and round glass gaskets (R = 0.5 cm, Guluo), and the upper-mechanical transfer device converts the finger pressure into the strain energy of the spring. Next, it is introduced into the hydrogel actuators to deform the RBCs consistently.

For Community Health Service Centers (CHSC): in light of the nosocomial infection, we performed a separate design (Fig. S1, left) for the smartphone and finger-press type microfluidics/imaging system, and we used a USB link to power the imaging system and transfer images. The finger-press type microfluidics/imaging system comprises a camera module (CMOS IMX214, RERVISION), LED module (SMD 5730, Goodlay), external optics lens, USB module, microfluidic chip, and mechanical transfer device.

**
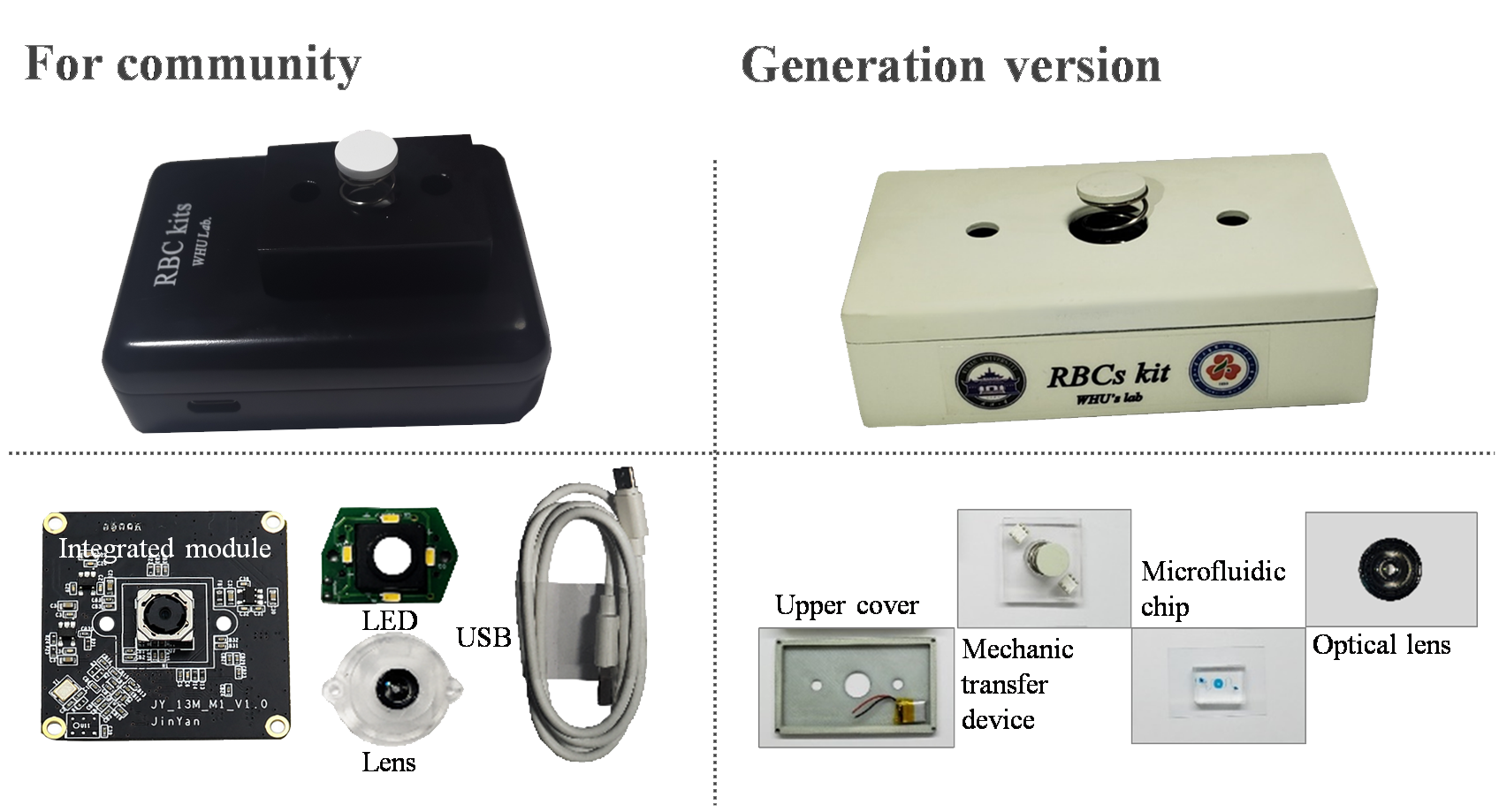
Supplementary Figures**

**Fig. S1. | Components of OPOC system and each test cost.**

|  | Hydrogel precursor | Fingertip blood collection needle | Micro blood collection pipette | Filin film | PDMS chip | Total |
| --- | --- | --- | --- | --- | --- | --- |
| For each test | < $0.3 | < $0.1 | < $0.1 | < $0.1 | < $0.4 | < $1.0 |

**Fig. S2. | Micrograph of the micro-particles (3 μm) based on this system, scale bar: 20 μm.**

**
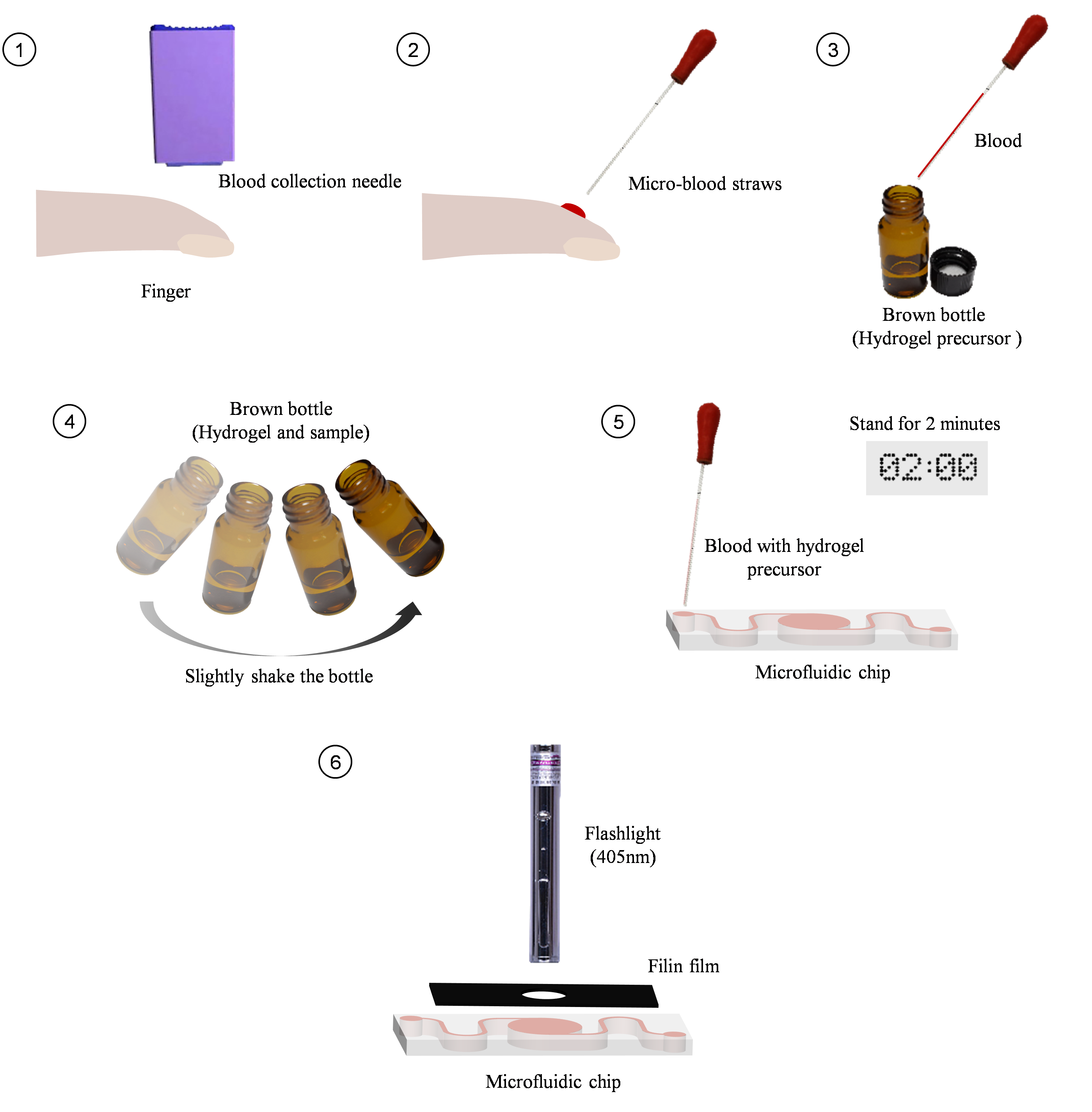
**

**Fig. S3. | Sample extraction and photo-curable hydrogel actuators.**

**
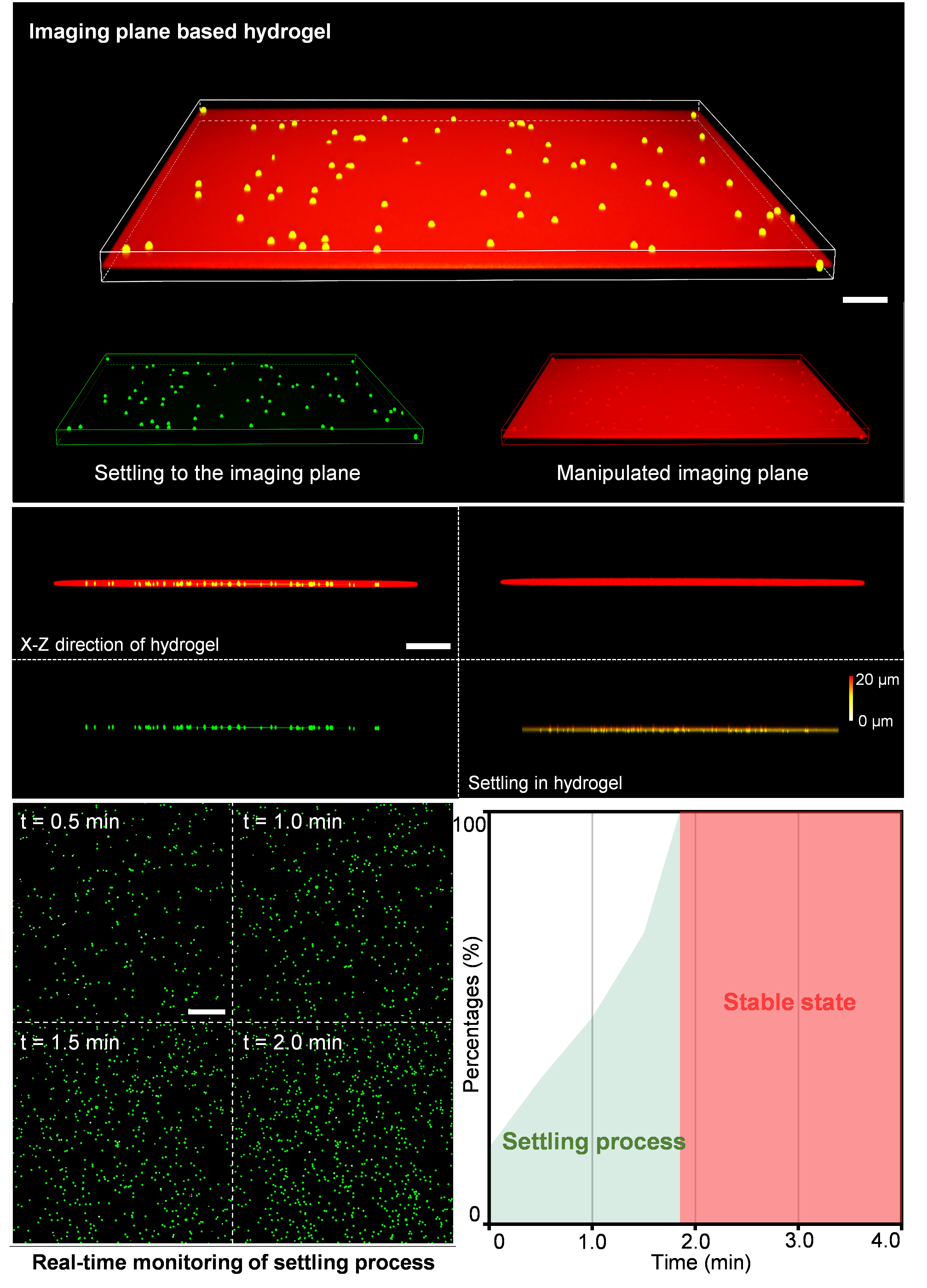
**

**Fig. S4. | Cell imaging plane based hydrogel, scale bar: 100 μm. X-Z direction of hydrogel, scale bar: 50 μm. Real-time monitoring of settling process (green fluorescent particle, 8μm), scale bar: 100 μm.**

**
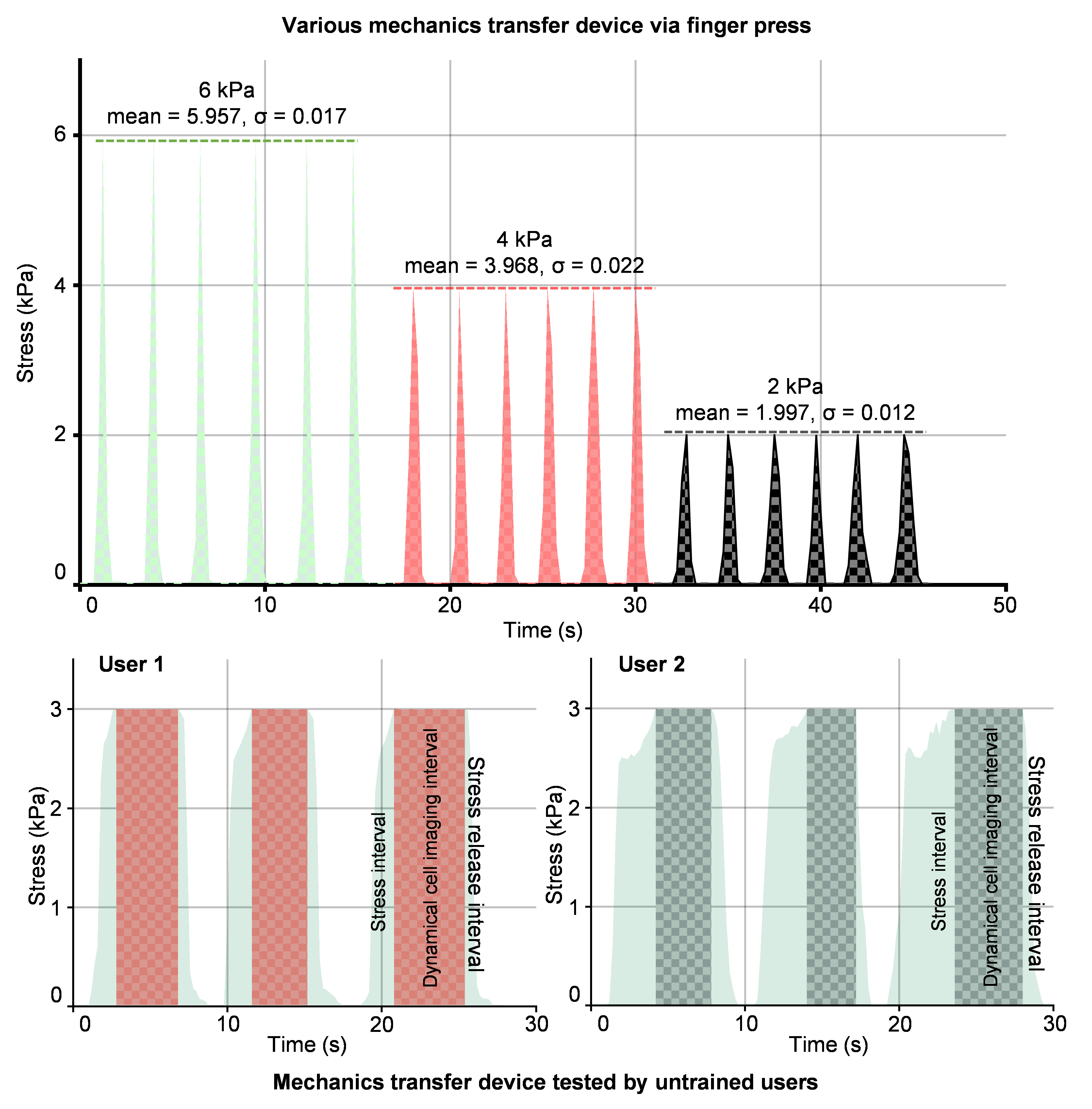
**

**Fig. S5. | Various mechanical transfer device via finger press. Mechanical transfer device tested by untrained users.**

**
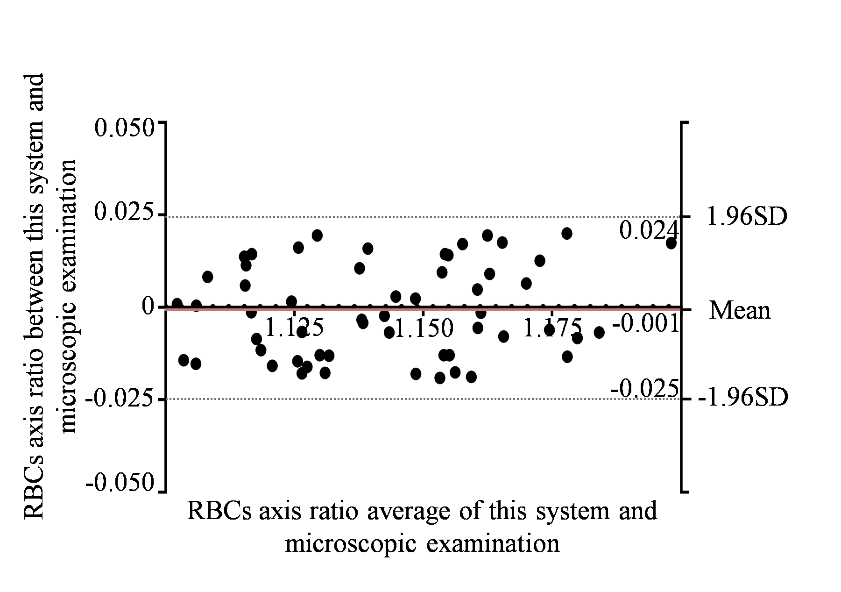
**

**Fig. S6. | Bland–Altman analysis to compare the mean axis ratio obtained by this system and microscopic examination.**

**
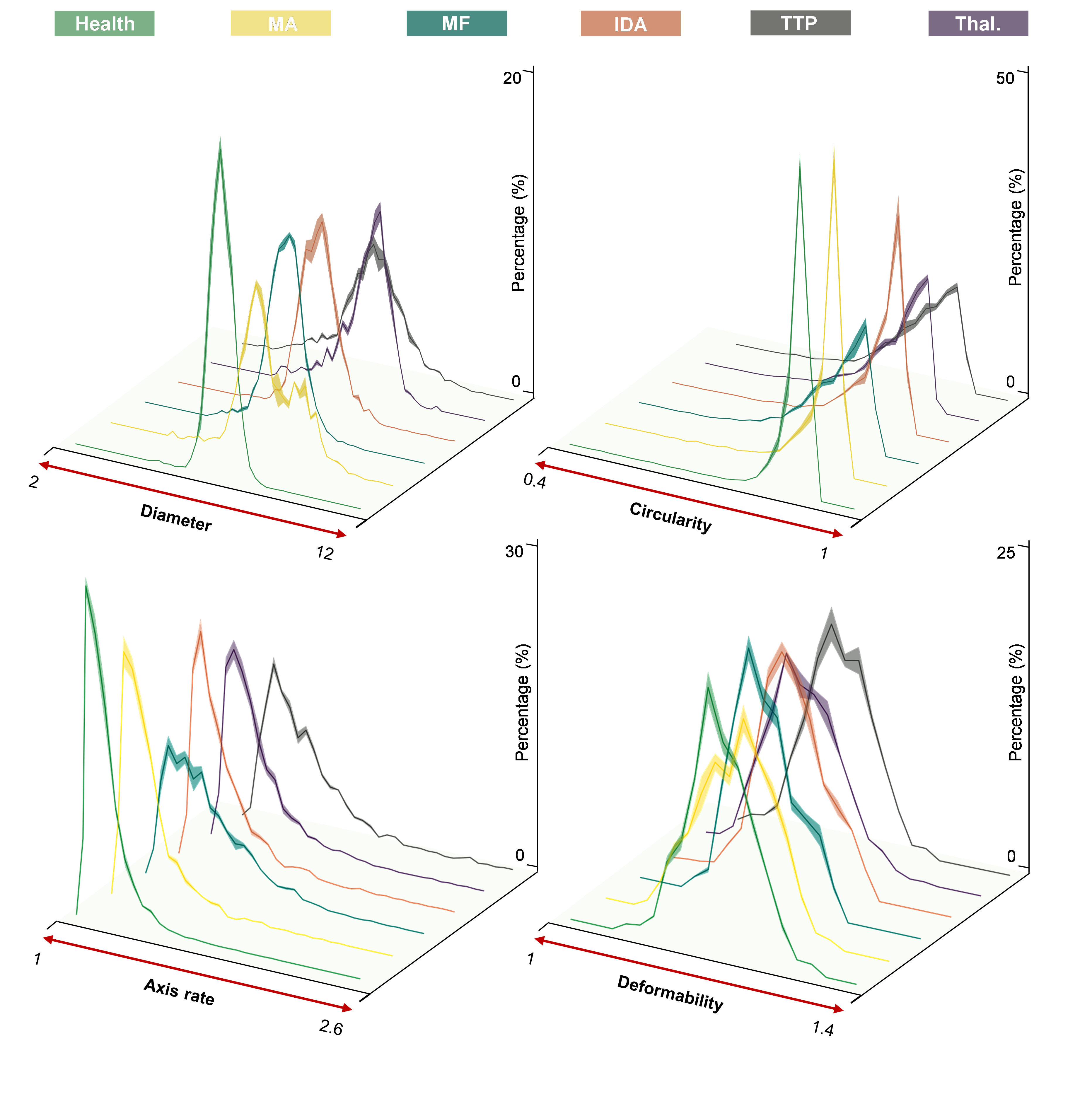
**

**Fig. S7.** **| Distributions of diameter, circularity, axis ratio, and deformability of 84 Health, 90 MA, 78 MF, 84 IDA, 48 TTP, and 48 Thal. participants from different patient donors.**

**a**

**Bone marrow**

**Marrow biopsy**

**Peripheral blood smear**

**Fig. S8. | Hospital pathology of six typical participants (a-f).**

Abbreviations follow those from Fig. S8: BM, bone marrow; MB, marrow biopsy; RBC, Red blood cell; NA, not available; CBC, complete blood cell; HGB, Hemoglobin; HCT, Hematocrit; MCV, mean red blood cell volume; MCH, Average hemoglobin content; MCHC, Mean hemoglobin concentration; RDW, Red blood cell distribution width.


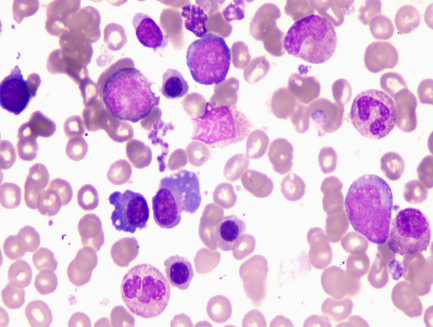

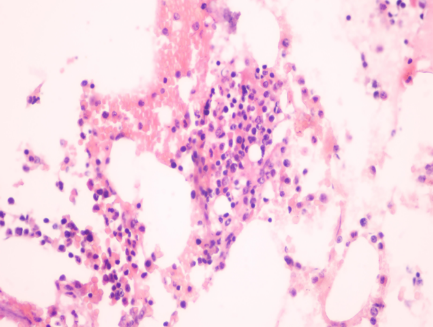

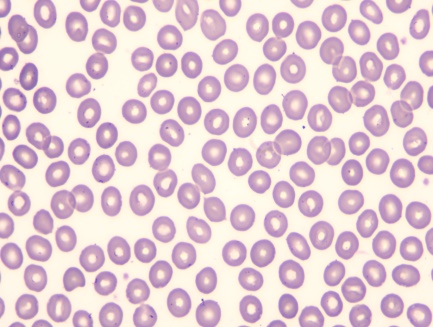


52 year old (y.o.) Female, **Healthy person**

**bone marrow (BM,×1000)**: Proliferation in erythroid, granular, megakaryotic series; **marrow biopsy(MB,×400):** Roughly normal; **peripheral RBCs smear (×1000):** normal.

**Genetic test**: No abnormality

**Analysis of CBC count**: RBC 4.11×10^12^/L, HGB 121 g/L, HCT 36.5%, MCV 88.8 fl, MCH 29.3 pg, MCHC 330 g/L, RDW 13%

**b**

**Bone marrow**

**Marrow biopsy**

**Peripheral blood smear**


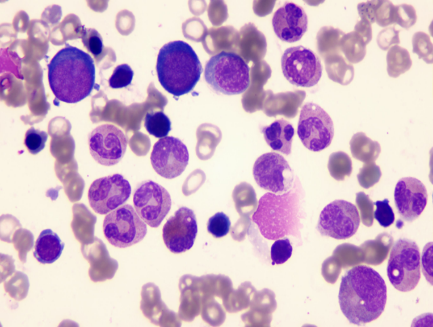

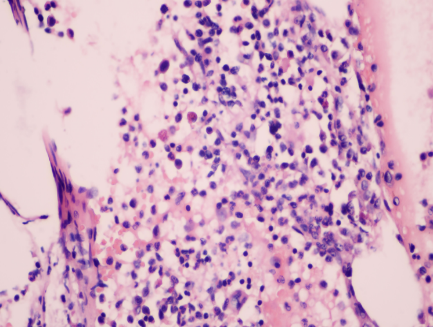

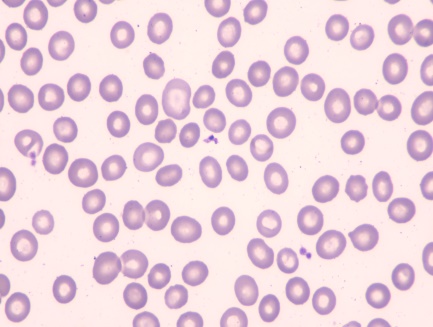


68 y.o. Male, **Iron deficiency anemia**

**BM:** Three-series hyperplasia with granular infection change, the proportion of red line increased, iron staining: inner iron (0%), outer iron (-); **MB:** Three-series hyperplasia, and increased proportion of erythroid; **peripheral RBCs smear:** Decreased volume, different sizes, and the central lightly stained area increased.

**Genetic test:** NA

**Analysis of CBC count:** RBC 2.9×1012/L, HGB 53 g/L, HCT 18.3%, MCV 63.1 fl, MCH 18.3 pg, MCHC 290 g/L, RDW 19.2%


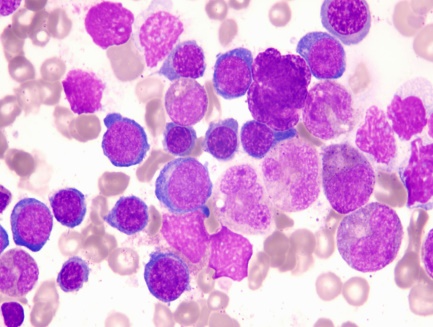

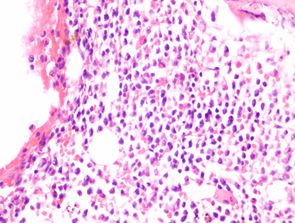

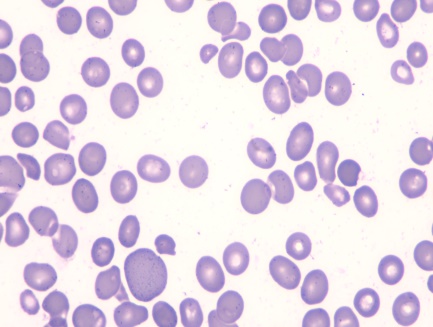


58 y.o. Female, **Megaloblastic anemia**

**BM:** increased erythron, granular series accompanied by megaloblast; the maturation of the grains is obstructed; inner iron (12%), outer iron (+); **MB:** Obviously active hyperplasia, and increased ratio of erythroid accompanied by megaloblast; **peripheral RBCs smear:** Increased volume, different sizes, and the central lightly stained area decreases, even disappears.

**Genetic test:** NA

**Analysis of CBC count:** RBC 1.79×10^12^/L, HGB 67 g/L, HCT 19.4%, MCV 108.4 fl, MCH 37.4 pg, MCHC 345 g/L, RDW 26%

**Marrow biopsy**

**Peripheral blood smear**

**Bone marrow**

**c**

56 y.o. Male, **Primary myelofibrosis**

**BM:** Three-series hyperplasia accompanied by granular infection changes, and platelets tend to be more; **MB:** Megakaryocyte proliferation accompanied by an increase in atypical megakaryocytes, with grade 3 reticular fibrosis seen; **peripheral RBCs smear:** Erythrocytes vary in size; teardrop cells are easy to see (account for 13.1%).

**Genetic test:** WT1,JAK2,ASXL1,TET2 and IDH1 gene mutation

**Analysis of CBC count:** RBC 2.8×10^12^/L, HGB 80 g/L, HCT 25.1%, MCV 89.5 fl, MCH 28.6 pg, MCHC 319 g/L, RDW 21.5%


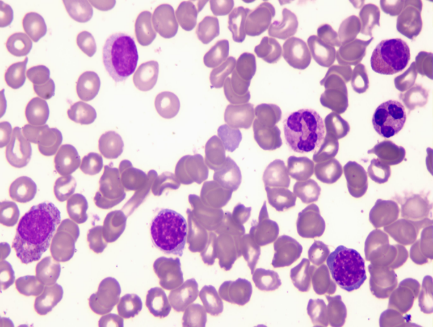

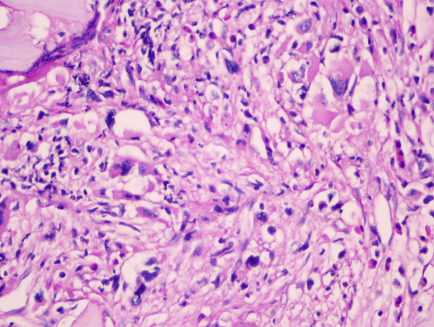

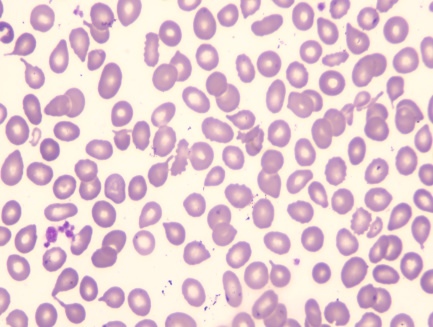


**Bone marrow**

**Peripheral blood smear**

**Marrow biopsy**

**d**

**Marrow biopsy**

**Bone marrow**

**Peripheral blood smear**


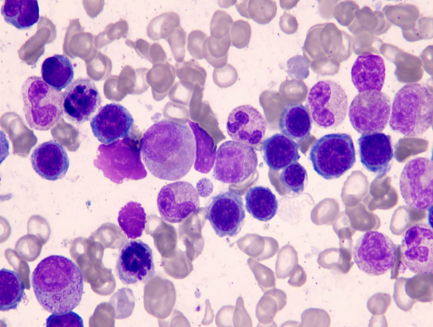

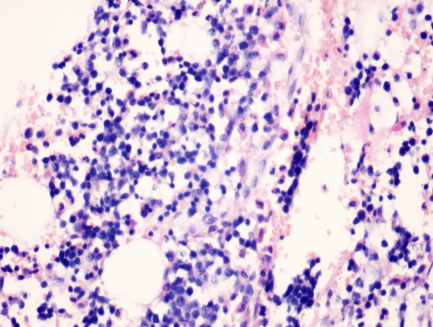

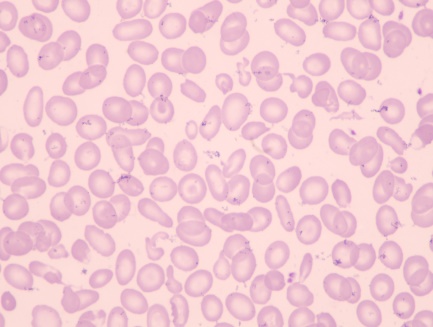


16 y.o. Female, **Alpha thalassemia**

**BM:** Three-series hyperplasia accompanied by granular infection changes, and the proportion of erythroid increases; **MB:** Hyperplasia is roughly normal, and the proportion of three-series hyperplasia with erythroid is significantly increased; **peripheral RBCs smear:** Decreased volume, different sizes, the central lightly stained area increased, and codocytes are easy to see.

**Genetic test:** --SEA and -α3.7 double deletion

**Analysis of CBC count:** RBC 3.44×10^12^/L, HGB 66.5 g/L, HCT 21.4%, MCV 62.1 fl, MCH 19.3 pg, MCHC 311.4 g/L, RDW 18.2%

**e**

66 y.o. Female, **Thrombotic Thrombocytopenic Purpura**

**BM:** Three-series hyperplasia with megakaryocyte dysplasia; **MB:** Hyperplasia is normal, and the proportion of three-series hyperplasia with erythroid is slightly increased; **peripheral RBCs smear:** Erythrocytes vary in size, and a large number of schizocytes can be seen.

**Genetic test:** No ADAMTS13 mutation detected.

**Analysis of CBC count:** RBC 3.28×10^12^/L, HGB 107 g/L, HCT 31.1%, MCV 94.8 fl, MCH 32.6 pg, MCHC 344 g/L, RDW 13.5%


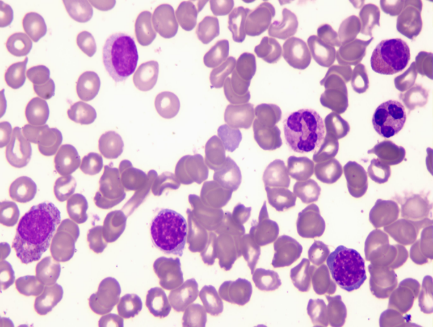

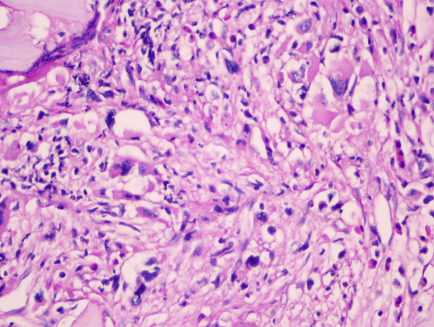

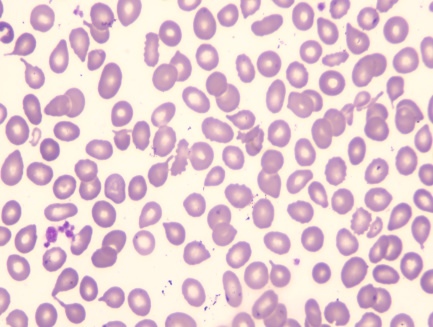


**Bone marrow**

**Peripheral blood smear**

**Marrow biopsy**

**f**

**
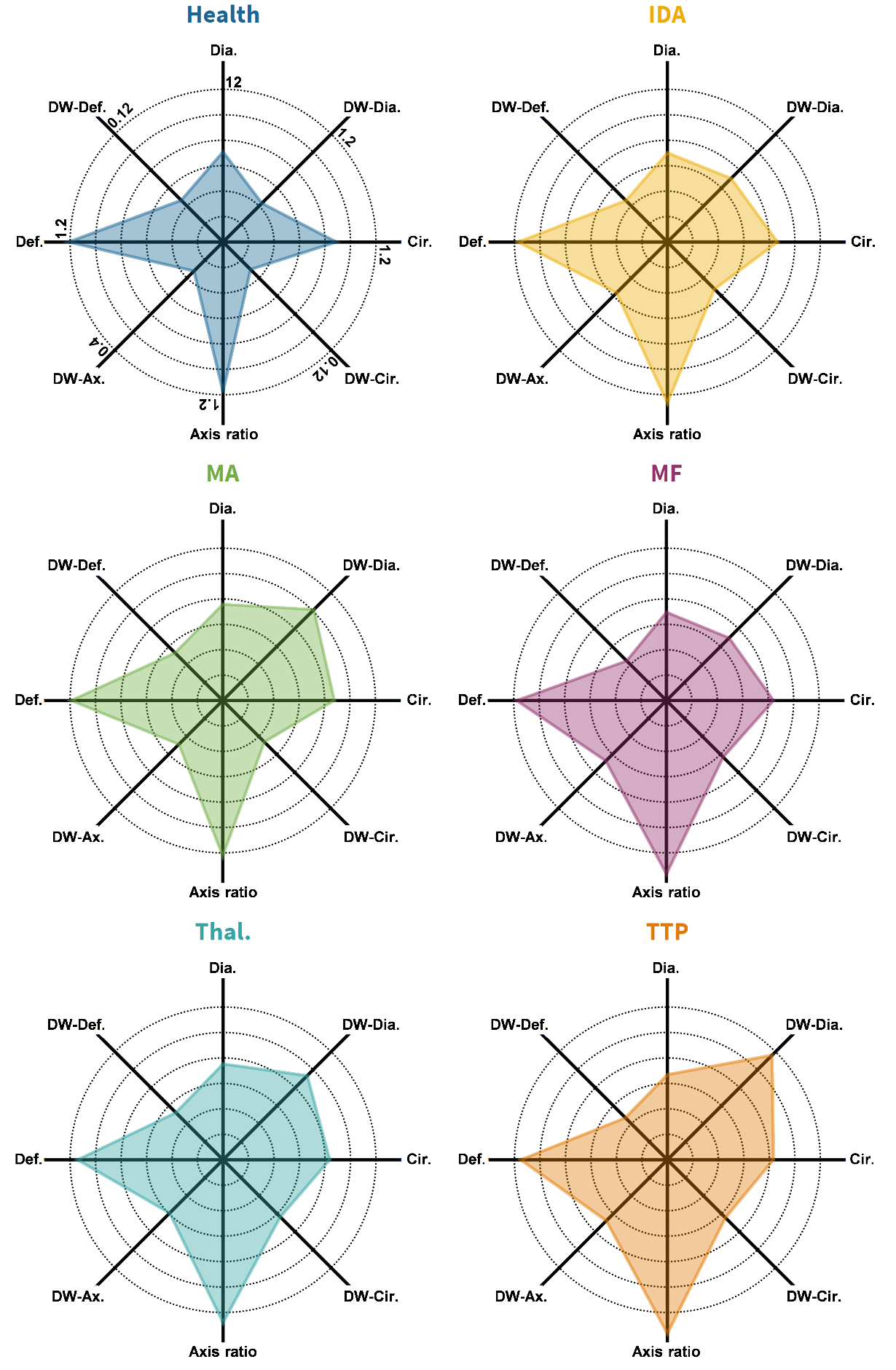
**

**Fig. S9. | Radar map of six typical participants with morphologic and mechanic parameters distribution.**

Abbreviations follow those from Fig. S9: Dia., diameter; DW-Dia., distribution width of diameter; Cir., circularity; DW-Cir., distribution width of circularity; DW-Ax., distribution width of axis ratio; Def., deformability; DW-Def., distribution width of deformability.

**Supplementary Tables**

**Table S1.** Patient admission pathology report of six typical participants after intelligent diagnosis.

| **Patient No.** | **1** | **2** | **3** | **4** | **5** | **6** |
| --- | --- | --- | --- | --- | --- | --- |
| Age (Year) | 52 | 68 | 58 | 56 | 16 | 66 |
| diagnosis | Healthy person | Iron deficiency anemia | Megaloblastic anemia | Primary myelofibrosis | Alpha thalassemia | Thrombotic thrombocytopenic purpura |
| Red blood cell morphology | Normal | Decreased volume, different sizes, and the central lightly stained area increased. | Increased volume, different sizes, and the central lightly stained area decreases, even disappears. | Erythrocytes vary in size; teardrop cells are easy to see (account for 13.1%). | Decreased volume, different sizes, the central lightly stained area increased, and codocytes are easy to see. | Erythrocytes vary in size, and a large number of schizocytes can be seen. |
| Bone marrow | Proliferation in erythroid, granular, megakaryotic series (three-series). | Three-series hyperplasia with granular infection change, the proportion of red line increased, iron staining: inner iron (0%), outer iron (-). | Increased erythron, granular series accompanied by megaloblast; the maturation of the grains is obstructed; inner iron (12%), outer iron (+). | Three-series hyperplasia accompanied by granular infection changes, and platelets tend to be more. | Three-series hyperplasia accompanied by granular infection changes, and the proportion of erythroid increases. | Three-series hyperplasia with megakaryocyte dysplasia. |
| Bone marrow biopsy | Roughly normal | Three-series hyperplasia, and increased proportion of erythroid. | Obviously active hyperplasia, and increased ratio of erythroid accompanied by megaloblast | Megakaryocyte proliferation accompanied by an increase in atypical megakaryocytes, with grade 3 reticular fibrosis seen. | Hyperplasia is roughly normal, and the proportion of three-series hyperplasia with erythroid is significantly increased. | Hyperplasia is normal, and the proportion of three-series hyperplasia with erythroid is slightly increased. |
| Genetic test | No abnormality. | NA | NA | WT1,JAK2,ASXL1,TET2, IDH1 gene mutation | --SEA and -α3.7 double deletion | No ADAMTS13 mutation detected. |
| RBC(10^12^/L) | 4.11 | 2.9 | 1.79 | 2.8 | 3.44 | 3.28 |
| HGB(g/L) | 121 | 53 | 67 | 80 | 66.5 | 107 |
| HCT(%) | 36.5 | 18.3 | 19.4 | 25.1 | 21.4 | 31.1 |
| MCV(fl) | 88.8 | 63.1 | 108.4 | 89.5 | 62.1 | 94.8 |
| MCH(pg) | 29.3 | 18.3 | 37.4 | 28.6 | 19.3 | 32.6 |
| MCHC(g/L) | 330 | 290 | 345 | 319 | 311.4 | 344 |
| RDW(%) | 13 | 19.2 | 26 | 21.5 | 18.2 | 13.5 |

**Table S2.** Mean diameter, circularity, axis ratio, and deformability of the 30 typical participants with this device.

| **Samples** | **Diameter** | **Circularity** | **Axis ratio** | **Deformability** |
| --- | --- | --- | --- | --- |
|  |  |  |  |  |
| **Case 1 (Health)** | 7.1064  (SD = 0.4325) | 0.8921  (SD = 0.0306) | 1.1829  (SD = 0.1078) | 1.2159  (SD = 0.0465) |
| **Case 2** | 7.1658  (SD = 0.4068) | 0.8827  (SD = 0.0261) | 1.1666  (SD = 0.1230) | 1.2124  (SD = 0.0410) |
| **Case 3** | 7.1387  (SD = 0.4216) | 0.8936  (SD = 0.0295) | 1.1573  (SD = 0.1176) | 1.2096  (SD = 0.0435) |
| **Case 4** | 7.1863  (SD = 0.4208) | 0.8916  (SD = 0.0329) | 1.1605  (SD = 0.1318) | 1.2165  (SD = 0.0446) |
| **Case 5** | 7.1236  (SD = 0.4384) | 0.8872  (SD = 0.0316) | 1.1658  (SD = 0.1192) | 1.2163  (SD = 0.0427) |
| **Case 1 (IDA)** | 7.0194  (SD = 0.7082) | 0.8657  (SD = 0.0524) | 1.2671  (SD = 0.1883) | 1.1742  (SD = 0.0462) |
| **Case 2** | 6.8541  (SD = 0.7369) | 0.8272  (SD = 0.0616) | 1.2358  (SD = 0.2092) | 1.1632  (SD = 0.0508) |
| **Case 3** | 6.7375  (SD = 0.7542) | 0.8365  (SD = 0.0563) | 1.2732  (SD = 0.2015) | 1.1716  (SD = 0.0487) |
| **Case 4** | 6.8112  (SD = 0.7369) | 0.8463  (SD = 0.0629) | 1.2569  (SD = 0.1968) | 1.1657  (SD = 0.0513) |
| **Case 5** | 6.9465  (SD = 0.7193) | 0.8337  (SD = 0.0583) | 1.2732  (SD = 0.2015) | 1.1683  (SD = 0.0479) |
| **Case 1 (MA)** | 7.5962  (SD = 1.0156) | 0.8793  (SD = 0.0464) | 1.2284  (SD = 0.1631) | 1.1956  (SD = 0.0532) |
| **Case 2** | 7.452  (SD = 1.0783) | 0.8939  (SD = 0.0493) | 1.1951  (SD = 0.1575) | 1.1886  (SD = 0.0628) |
| **Case 3** | 7.571  (SD = 1.0937) | 0.8912  (SD = 0.0436) | 1.2043  (SD = 0.1549) | 1.1923  (SD = 0.0667) |
| **Case 4** | 7.4399  (SD = 1.0338) | 0.8827  (SD = 0.0448) | 1.1865  (SD = 0.1679) | 1.2071  (SD = 0.0568) |
| **Case 5** | 7.4289  (SD = 1.1106) | 0.8796  (SD = 0.0428) | 1.2167  (SD = 0.1646) | 1.1985  (SD = 0.0603) |
| **Case 1 (MF)** | 6.9689  (SD = 0.6983) | 0.8434  (SD = 0.0632) | 1.3689  (SD = 0.2249) | 1.1796  (SD = 0.0443) |
| **Case 2** | 6.8416  (SD = 0.7161) | 0.8612  (SD = 0.0655) | 1.3341  (SD = 0.2423) | 1.1721  (SD = 0.0469) |
| **Case 3** | 6.7116  (SD = 0.6694) | 0.8446  (SD = 0.0691) | 1.3002  (SD = 0.2484) | 1.1809  (SD = 0.0506) |
| **Case 4** | 6.8571  (SD = 0.6374) | 0.8501  (SD = 0.0612) | 1.3216  (SD = 0.2356) | 1.1832  (SD = 0.0478) |
| **Case 5** | 6.7759  (SD = 0.6875) | 0.8552  (SD = 0.0602) | 1.2968  (SD = 0.2273) | 1.1767  (SD = 0.0432) |
| **Case 1 (Thal.)** | 7.5456  (SD = 0.9361) | 0.8389  (SD = 0.0637) | 1.2826  (SD = 0.1981) | 1.1453  (SD = 0.0525) |
| **Case 2** | 7.5641  (SD = 0.8971) | 0.8148  (SD = 0.0585) | 1.2949  (SD = 0.2051) | 1.1534  (SD = 0.0509) |
| **Case 3** | 7.5354  (SD = 0.9075) | 0.8411  (SD = 0.0607) | 1.2916  (SD = 0.2025) | 1.1552  (SD = 0.0547) |
| **Case 4** | 7.5049  (SD = 0.9151) | 0.8446  (SD = 0.0591) | 1.3045  (SD = 0.2201) | 1.1486  (SD = 0.0507) |
| **Case 5** | 7.5126  (SD = 0.9198) | 0.8232  (SD = 0.0598) | 1.3002  (SD = 0.2077) | 1.1513  (SD = 0.0522) |
| **Case 1 (TTP)** | 6.7023  (SD =1.1635) | 0.8333  (SD = 0.0645) | 1.3671  (SD = 0.2262) | 1.1522  (SD = 0.0467) |
| **Case 2** | 6.7378  (SD = 1.1507) | 0.8298  (SD = 0.0606) | 1.3576  (SD = 0.2281) | 1.1486  (SD = 0.0453) |
| **Case 3** | 6.6826  (SD = 1.1816) | 0.8177  (SD = 0.0617) | 1.3638  (SD = 0.2543) | 1.1443  (SD = 0.0437) |
| **Case 4** | 6.7457  (SD = 1.1721) | 0.8538  (SD = 0.0631) | 1.3195  (SD = 0.2095) | 1.1463  (SD = 0.0476) |
| **Case 5** | 6.6249  (SD = 1.1863) | 0.8356  (SD = 0.0598) | 1.3521  (SD = 0.2112) | 1.1497  (SD = 0.0466) |

| **Blood samples (Storage days)** | **Diameter** | **Circularity** | **Axis ratio** | **Deformability** |
| --- | --- | --- | --- | --- |
|  |  |  |  |  |
| **15** | 6.6203  (SD = 0.5155) | 0.8303  (SD = 0.0576) | 1.1622  (SD = 0.1572) | 1.2036  (SD = 0.0536) |
| **16** | 6.6354  (SD = 0.5180) | 0.8223  (SD = 0.0601) | 1.1626  (SD = 0.1620) | 1.2016  (SD = 0.0512) |
| **15** | 6.6536  (SD = 0.5142) | 0.8358  (SD = 0.0583) | 1.1608  (SD = 0.1638) | 1.1956  (SD = 0.0565) |

**Table S3.** Mean diameter, circularity, axis ratio, and deformability of the misdiagnosis samples for blood quality.
