# Supplementary material for: Touchable cell biophysics property recognition platforms enable multifunctional blood smart health care
Source: Microsyst Nanoeng. 2021 Dec 8;7:103. doi: 10.1038/s41378-021-00329-z (PMC8651774; doi:10.1038/s41378-021-00329-z)
Supplement: Supplementary file 2 — Ethical approve [file 41378_2021_329_MOESM2_ESM.pdf]

武汉大学中南医院医学伦理委员会科研项目伦理审批件
Ethical Approval for Clinical/Scientific Research Projects under Medical Ethics Committee, Zhongnan Hospital of Wuhan University

批件号：科伦[2020153]

<table>
<tr><td>项目名称
Project Name</td><td colspan="4">基于时域拉伸超快光学成像技术的血细胞分析
Blood cell analysis based on time-domain stretched ultrafast optical imaging technique</td></tr>
<tr><td>申办者
Sponsor</td><td colspan="4">武汉大学中南医院
Zhongnan Hospital of Wuhan University</td></tr>
<tr><td>科室
Department</td><td colspan="2">血液内科
Hematology department</td><td>主要研究者
Primary Investigator</td><td>周芙玲
Fulin Zhou</td></tr>
<tr><td>项目来源
Project Resource</td><td colspan="4">武汉大学中南医院临床研发项目
Clinical Research and development project of Zhongnan Hospital of Wuhan University</td></tr>
<tr><td>审核清单
The List of Reviewed Documents</td><td colspan="4">1. 伦理审查申请表 Ethics review application form
2. 研究者简历 Researcher resume
3. 研究方案 Research plan（V1.0/2020.07.02）
4. 知情同意书 Informed consent（V1.0/2020.07.02）
5. 质量管理方案 Quality management plan
6. 项目涉及的伦理流程 The ethical process involved in the project</td></tr>
<tr><td>审查类型
review Category</td><td>☑初始审查 Primary　☐修正审查 Amendment</td><td>审批方式
Review Type</td><td colspan="2">☑会议审查 Conference　☐快速审查 Quick</td></tr>
<tr><td>日期 Date</td><td>2020-07-12</td><td>地点 Place</td><td colspan="2">门诊 13 楼 2 号会议室
No. 2 conference room, 13th floor, outpatient clinic</td></tr>
<tr><td>审查意见
Review Opinion</td><td colspan="4">1. 经本伦理委员会审查投票，伦理委员会意见：同意。
2.论文发表请按照我院科研处的相关程序报批。
For the publication of papers, please follow the relevant procedures of our scientific research department for approval.
3.伦理批件有效期：12 个月。
Validity period: 12 months .
主任委员或副主任委员或授权人签字：
Signature of Chairman or Vice Chairman:
武汉大学中南医院医学伦理委员会（盖章）
Medical Ethics Committee
Zhongnan Hospital of Wuhan University（Stamp）
日期 Date: 2020-07-14</td></tr>
<tr><td colspan="5">注意事项 Notice:</td></tr>
</table>

1. 请遵循 NMPA/GCP 原则和《赫尔辛基宣言》、遵循本伦理委员会批准的方案开展临床研究，保护受试者的健康与权利。

Please comply with NMPA / GCP Principles and Helsinki Declaration, and carry out clinical trials according to code of ethics approved by our medical ethics committee in protection of the health and rights of human subjects

2.研究过程中，对研究方案和知情同意书等相关文件的修改，均须得到伦理委员会审查同意后方可实施。

All paperwork modification related to research proposal and informed consent during the course of study are subject to approval of the Ethics Committee.

3.发生严重不良事件或影响研究风险受益比的非预期不良事件、违背方案、暂停/终止研究须及时报告本伦理委员会。

Report timely to the medical ethics committee on incidents with serious adverse impact, unexpected incidents negatively affecting risk benefit ratio, breach of program or suspension / termination of study.

4.根据项目来源类别提交跟踪审查材料。 Submit follow-up review paperwork based on project resource categories

5.凡是涉及人类遗传资源出口或按照国家规定必须经有关部门审批的内容，均需在项目执行前向有关部门申报并获得批准。非以产品注册为目的临床研究需在医学研究登记备案信息系统(：http://114.255.48.20)注册后方可研究。

All contents regarding exporting of human genetic resources or approval by responsive Departments of Government, should be applied and approved in advance.Clinical research not for product registration needs to be registered in the Medical Research Registration Filing Information System (http://114.255.48.20) before research.

伦理委员会声明 Statement：
本伦理委员会严格按照中国 GCP 及相关法律法规组成及工作。
The Ethics Committee is well organized and works strictly in accordance with the Chinese GCP and related laws and regulations.

伦理委员会地址：湖北省武汉市武昌区东湖路 169 号门诊楼 9 楼
Address: Outpatient building, 9 floor, No.169 Donghu Road, Wuchang District, Wuhan, Hubei, China
邮编 Post Code：430071； 电话 Phone：+86-27-67812787

武汉大学中南医院医学伦理委员会

武汉大学中南医院医学伦理委员会成员名单及出席情况

Committee member attendance sheet of medical ethics committee, Zhongnan hospital of Wuhan University

时间：2020-07-12　　14:00　　　　地点：门诊 13 楼 2 号会议

序号 Number	姓名 Name	性别 Gender	专业 Speciality	单位 Work Unit	签名 Signature
1	张元珍* Yuanzhen Zhang	女	妇产科学 Gynaecology	武汉大学中南医院 Zhongnan Hospital	Yuanzhen Zhang
2	周福祥# Fuxiang Zhou	男	肿瘤学 Oncology	武汉大学中南医院 Zhongnan Hospital	
3	喻爱喜# Aixi Yu	男	骨外科学 Bone surgery	武汉大学中南医院 Zhongnan Hospital	Aixi Yu
4	林军 Jun Lin	男	消化内科学 Gastroenterology	武汉大学中南医院 Zhongnan Hospital	林军 Jun Lin
5	李志强 Zhiqiang Li	男	神经外科学 Neurosurgery	武汉大学中南医院 Zhongnan Hospital	缺席
6	蒋明 Ming Jiang	男	伦理学 Ethics	武汉大学医学部 Wuhan University School of Medicine	
7	尹平 Ping Yin	男	生物统计学 Biostatistics	华中科技大学同济医学院 Tongji Medical College	
8	王有为 Youwei Wang	男	中药学 Chinese Pharmacology	武汉大学药学院 College of pharmacy	
9	黄建英 Jianying Huang	女	新药临床评价 Clinical evaluation of new drugs	武汉大学中南医院 Zhongnan Hospital	Jianying Huang
10	王扬淦 Yanggan Wang	男	心血管内科学 Cardiology	武汉大学中南医院 Zhongnan Hospital	
11	徐海波 Haibo Xu	男	医学影像与核医学 Imaging and Nuclear Medicine	武汉大学中南医院 Zhongnan Hospital	
12	李一荣 Yirong Li	男	医学检验学 Medical Laboratory Science	武汉大学中南医院 Zhongnan Hospital	
13	吴东方 Dongfang Wu	男	药学 Pharmacology	武汉大学中南医院 Zhongnan Hospital	
14	彭志勇 Zhiyong Peng	男	重症医学 Critical care	武汉大学中南医院 Zhongnan Hospital	
15	汪春红 Chunhong Wang	女	流行病学 Epidemiology	武汉大学健康学院 Health college	Wang chunhong
16	陈璐瑄 Luxuan Chen	女	思想政治教育 Political education	社区群众 The community crowd	Luxuan Chen
17	张粒 Li Zhang	女	法学 Law	北京中伦律师事务所 Beijing zhonglun law firm	Li Zhang
18	孙才华 Caihua Sun	男	法学 Law	北京中伦律师事务所 Beijing zhonglun law firm	

* 主任委员　Chairman，# 副主任委员　Associate Chairman

注：律师为 AB 角色，每次伦理会一人参会，上述人员名单从 2019 年 8 月 5 日生效。
